# High-Caloric and Chocolate Stimuli Processing in Healthy Humans: An Integration of Functional Imaging and Electrophysiological Findings

**DOI:** 10.3390/nu6010319

**Published:** 2014-01-10

**Authors:** Deyar Asmaro, Mario Liotti

**Affiliations:** Department of Psychology, Simon Fraser University, 8888 University Drive, RCB 5246, Burnaby, BC V5A 1S6, Canada; E-Mail: mliotti@sfu.ca

**Keywords:** chocolate, fMRI, ERPs, high-caloric

## Abstract

There has been a great deal of interest in understanding how the human brain processes appetitive food cues, and knowing how such cues elicit craving responses is particularly relevant when current eating behavior trends within Westernized societies are considered. One substance that holds a special place with regard to food preference is chocolate, and studies that used functional magnetic resonance imaging (fMRI) and event-related potentials (ERPs) have identified neural regions and electrical signatures that are elicited by chocolate cue presentations. This review will examine fMRI and ERP findings from studies that used high-caloric food and chocolate cues as stimuli, with a focus on responses observed in samples of healthy participants, as opposed to those with eating-related pathology. The utility of using high-caloric and chocolate stimuli as a means of understanding the human reward system will also be highlighted, as these findings may be particularly important for understanding processes related to pathological overeating and addiction to illicit substances. Finally, research from our own lab that focused on chocolate stimulus processing in chocolate cravers and non-cravers will be discussed, as the approach used may help bridge fMRI and ERP findings so that a more complete understanding of appetitive stimulus processing in the temporal and spatial domains may be established.

## 1. Introduction

Scientific attention towards understanding how people process appetitive food cues has seen a major raise over the course of the last decade, partly due to the increased interest that has been devoted to understanding disordered eating. Researchers who use neuroimaging techniques have attempted to identify the neural correlates of appetitive food processing, and by using functional neuroimaging and electroencephalography (EEG), brain activations elicited by presentations of highly appetitive food cues have been identified in the temporal and spatial domains.

Collectively, a large number of studies have been carried out using functional magnetic resonance imaging (fMRI) and event-related potentials (or ERPs, which are extracted from EEG recordings). Both of these techniques have inherent advantages and disadvantages, with fMRI providing excellent spatial resolution due to its measurement of BOLD (blood oxygen level-dependent) changes within key regions of the brain during experimental tasks. However, because such effects happen over a matter of seconds, the fMRI technique is thought to have relatively poor temporal resolution. Ultimately, this limitation restricts the method’s ability to address certain research questions (*i*.*e*., what cognitive processes are recruited within 200–300 ms after a chocolate image is presented?). Additionally, it is very expensive to acquire, operate, and maintain fMRI equipment, thereby limiting the number of studies that could be conducted within a given period of time. On the other hand, ERPs have very high temporal resolution and can be used to document changes in brain electrical activity recorded over the scalp on a millisecond-by-millisecond basis due to the fact electrical responses travel at nearly the speed of light and reach external sensors rapidly. Because of this property, the technique is especially well suited for identifying the bottom–up processes that underlie phenomena such as reward valuation and decision making, as well as subconscious processes that may underlie various automatically generated behaviors (all of which are relevant to food stimulus perception). Also, the equipment is relatively inexpensive and does not require the large amount of space needed for fMRI data collection. However, the spatial resolution of scalp-recorded EEG is less precise because electrical signals generated inside the brain reach the scalp though volume conduction, which reduces and smears the signal as distance from the source increases. In addition, distinct tissues (gray matter, fiber tracts, air/fluid, bone) conduct electricity at different rates, further complicating the precise localization of generators inside the brain. Brain source localization relies on complex source modeling techniques which attempt to identify where electrical signatures detected at the scalp come from by solving the so-called inverse problem. These have historically produced biased estimates that favor sources closer to the cortical surface, and have also had difficulty identifying activations that are produced in deeper brain tissue. One further methodological issue that is pertinent to the investigation of food cue processing in the brain relates to stimulus presentation techniques that can be utilized during a study. Using fMRI, it is possible to have a participant consume various foods while they complete a given task, as the small movements that are made within the scanner can be corrected during data processing. Unfortunately, these same movements create more serious artifacts when the ERP technique is used, therefore contaminating experimental trials within the research paradigm and affecting data quality. Despite the advantages and disadvantages inherent in the utilization of these methods, some researchers are now trying to compare and combine ERP and fMRI findings so that neural structures and cognitive processes implicated in various aspects of appetitive food stimulus processing can be identified and better understood.

A particularly appetitive substance that has been used in studies of food processing is chocolate. Research indicates that chocolate does indeed hold a special status as it elicits brain activity that is unique relative to other categories of foods [1] and recruits brain structures that also respond to craving-inducing stimuli (see [2,3]). Decreases in chocolate cue-related brain activity have also been observed after it is consumed to levels of satiety (*i*.*e*., [4,5]). Furthermore, using chocolate as a stimulus has allowed researchers to elicit craving responses and measure satiety effects using a unique and naturally rewarding substance. By using the unique properties of chocolate in food craving research, insights into drug addiction processes can also be made, which may shed light on clinically relevant issues that deal with this particular form of pathology.

This review will summarize findings that were reported by researchers who presented high-caloric food stimuli to samples of healthy participants. Findings that were obtained using fMRI and ERPs will be highlighted, and different sections of the paper will also specifically discuss fMRI and ERP effects discovered using multimodal presentations of chocolate stimuli. Finally, we will attempt to bridge these two areas of research by showcasing findings we obtained when we conducted an ERP study of chocolate stimulus processing that utilized a source localization technique (therefore facilitating a comparison between results obtained via electrophysiological recordings and those obtained while observing BOLD responses to chocolate cues). Before the relevant research is examined in detail, we will briefly review the neuroanatomy of the human gustatory and olfactory systems and the proposed functions for some neural areas that process stimuli presented through these sensory streams.

## 2. Neuroanatomy of the Gustatory and Olfactory Pathways

Researchers have made great advances in understanding how human beings process gustatory and olfactory cues. Advances in brain sciences have granted us unparalleled access into the inner workings of our nervous systems, and the pathways that process tastes and smells have been described in intricate detail. We will review some of the basic research that has been done in this area to provide a context for understanding results that were obtained in studies that specifically assessed reactivity to food cues that were presented to various sensory modalities.

### 2.1. The Neural Pathways Underlying Taste Perception (Gustation)

Taste signals begin when specialized chemosensory cells (taste buds) located in regions of the oral cavity (tongue and epithelium) are activated by a taste stimulus. These specialized cells respond preferentially to sweet, sour, bitter, salty, and umami stimulation (the five basic tastes) [6]. Once activated, the cells project efferent fibers to the central nervous system, whereby taste information is transmitted by cranial nerves to areas of the brain that process sensory inputs. Subcortical processing begins in the gustatory nucleus of medulla and proceeds to the thalamus, where gustatory information is routed to the primary taste cortex (consisting of the anterior insula and frontal operculum; [7]). From there, efferent outputs project to the amygdala, hypothalamus, dopaminergic areas of the midbrain, and secondary taste cortex (see [8]). Neurons at the level of the primary taste cortex are broadly tuned, meaning that they become activated by a number of tastes rather than specific ones (unlike what is observed at the level of the taste buds). Furthermore, neurons located in the secondary taste cortex (which is thought to be located in the orbitofrontal cortex (or OFC) and insula) are thought to perform multimodal integration processes, perhaps allowing for taste and smell information to be combined in order to create flavor [7,9,10]. The insula is also thought to be involved in interoceptive awareness (awareness of stimuli originating in the body), hedonic valuation, and may play a fundamental role in the expression of some eating disorders [11,12]. Importantly, the OFC also plays an important role in coding the reward value of a given food [13], which may also act in tandem with midbrain dopaminergic influences [14] to facilitate eating regulation (where “wanting” food changes with satiety).

### 2.2. The Neural Pathways Underlying Odor Perception (Olfaction)

Olfactory stimulation is first received by olfactory receptor neurons in the main olfactory epithelium, which transmit chemosensory signals to the olfactory bulb via their axonal projections. The reception of odor signals by olfactory receptor neurons differs from that seen with the reception of chemosensory stimulation by taste buds, as different odorants can be processed by the same olfactory neurons (therefore allowing for wide-ranging chemical discrimination by various neural ensembles) [15]. Once these signals reach the olfactory bulb, they are received by special nuclei called glomeruli. Olfactory stimulation continues towards higher neural regions by means of the olfactory tract, which is composed of efferent projections originating from cells that make up the glomeruli. The olfactory tract projects to the primary olfactory cortex, which is composed of several regions within the frontal and temporal lobes (primarily the piriform and entorhinal cortices) [16]. After being received by the primary olfactory cortex, olfactory signals reach the secondary olfactory cortex, including the insula and OFC, as well as the hypothalamus (which is implicated in the maintenance of homeostasis), and the amygdala (which is implicated in emotion appraisal). Perhaps because of the connections between efferent fibers from the olfactory bulb and structures such as the entorhinal cortex, OFC, amygdala, and insula, olfactory stimulation is known to elicit vivid recall of past memories and induction of various emotional states [17], and can also moderate subjective food craving [18].

## 3. High-Caloric Food Processing and the Human Brain

A number of studies have used both high-caloric and low-caloric foods as stimulus categories in attempts to understand how the brain responds to highly salient, craving-inducing stimuli. Findings from fMRI research indicate that a neural network is involved in the processing of such stimuli, and ERP research indicates that these cues recruit different nuclei during the early and late stages of stimulus processing.

### 3.1. fMRI Research

Perhaps not surprisingly, the majority of research done using high-caloric food stimuli in experimental paradigms with healthy participants has utilized fMRI. Although a large number of studies have looked at food stimulus processing in a general sense [12], only a subset have specifically looked at how high-caloric food stimuli are processed by healthy people (*i*.*e*., [19,20]). The following paragraphs will summarize recent fMRI findings and will highlight some of their implications.

#### 3.1.1. fMRI and High-Caloric Food Cue Reactivity

Research using high-caloric food images in conjunction with fMRI has been accomplished with a variety of experimental manipulations (see Table 1). High-caloric food stimuli have been found to produce unique activations relative to low-caloric food stimuli, with greater bilateral responses being observed in the medial prefrontal cortex (mPFC), dorsolateral prefrontal cortex (DLPFC), and thalamus during a task which required participants to try and remember what pictures they had previously viewed [19]. Differences in high-caloric food cue reactivity during satiated and fasted states have also been observed after similar cue presentation, with brain activations in the medial orbitofrontal cortex (mOFC) and bilateral amygdala being higher when participants fasted [20]. Interestingly, the mOFC activation disappeared once subjects were satiated, yet a right amygdala activation remained and was positively correlated with greater hunger ratings (potentially suggesting that the cue was still affectively relevant despite its reward value being diminished). Another study that used a fasting paradigm also found activations in the bilateral amygdala, anterior cingulate gyrus, hypothalamus, striatum, and visual processing areas in response to highly palatable food images [21]. In addition, a significant positive correlation was found between activation of the hypothalamus and levels of the hormone ghrelin that were recorded during fasting. Ghrelin levels were also found to be positively correlated with self-reported hunger during fasting and after caloric load conditions. Areas of the brain sensitive to reward valuation (e.g., the nucleus accumbens or NAcc) have also been shown to respond to appetitive food stimuli (food *versus* non-food objects), and were also found to be predictive of future snack consumption [22] and weight gain [23]. A recent meta-analysis also implicated the left amygdala, bilateral OFC, bilateral insula, and striatum in the processing of appetitive food cues [24]. Other research has also found that exercise significantly modulates BOLD responses to high-energy food cues, where reduced activity was observed in the insula when participants who completed an exercise routine were compared to a group that did not exercise [25]. Interestingly, completing exercise prior to viewing the high-energy food cues also increased neural activations to the sight of food in the left precuneus, which may reflect the operation of higher order cognitive functions that were engaged during the cueing task.

Looking at this body of research collectively, highly appetitive and/or high-caloric food cues appear to activate brain regions that are implicated in the appraisal of rewards and reward-based learning (striatum, mOFC), top-down regulation (anterior cingulate cortex or ACC, DLPFC), affect (amygdala), decision making (DLPFC, mPFC), interoceptive awareness and hedonic evaluation (insula) and homeostasis (hypothalamus) when they are viewed. Furthermore, it appears that activations observed within some of these regions are sensitive to subject-specific factors such as hunger level and dietary conscientiousness induced by exercise, with brain activations to the sight of this stimulus type being moderated by the coded value of externally and internally generated stimuli. By working in concert, these individual brain regions appear to enable a representation of stimulus value, which are presumably related to observable behavior. It is also significant that brain activations to the sight of these cues successfully predicted future weight gain and snack consumption. Future research in this direction could be extremely useful for researchers interested in clinical aspects of abnormal eating behavior, as well as those working in private industry who wish to better understand consumer populations.

#### 3.1.2. High-Caloric Food Cue Processing and Reward

Given that neural structures implicated in reward processing are consistently activated by the sight of appetitive food cues when healthy participants view them (*i*.*e*., [2,3,21,22,23,26,27,28,29,30,31,32]) and that many of the neural structures activated by the sight of food overlap with those activated when addicts view drug stimuli [33,34], understanding how reward circuitry in the brain is differentially activated by high-caloric relative to non-food and low-caloric stimuli appears to provide a novel way for researchers to better understand the neural basis of the human reward system. A recent meta-analysis found that a network of neural structures became activated during the processing of reward-related stimuli, which nicely demonstrates the intricate connections that exist between key dopaminergic centers in the brain (*i*.*e*., the nucleus accumbens, pallidum, thalamus) and prefrontal/limbic regions (including the anterior insula, lateral/medial OFC, lateral prefrontal cortex, hippocampus, amygdala, and ACC) [35]. Goldstone *et al*. (2009) found that there was greater activation in the ventral striatum, bilateral insula, medial and lateral OFC, and amygdala when participants who fasted overnight viewed high-caloric foods relative to a “fed” condition (indicating heightened responsiveness of the reward system due to the enhanced salience of food rewards) [29]. Several studies even found that neural responses to food items vary based on attractiveness of the particular stimulus, with highly appetitive, high-caloric foods eliciting greater activation of the amygdala and medial OFC when participants were hungrier [3,36]. Studies have also found that activation of the nucleus accumbens, a key structure where dopaminergic transmission facilitates the coding of reward anticipation (*i*.*e*., [37,38]), changes dramatically when healthy women view high-caloric and highly appetitive foods during different phases of the menstrual cycle, with greater right nucleus accumbens activation to high-caloric food cues during the follicular phase only [27]. Importantly, the OFC and DLPFC were activated by the sight of high-caloric food cues in both the follicular and luteal phases, an effect that resembles what Burger and Stice (2011) found when they administered chocolate milkshakes to adolescent women who were higher in trait eating restraint [39]. Regarding the effects of gender, one study also found that women were more responsive to the reward value of high-caloric food images than men were, with increased activations being observed in the dorsolateral, ventrolateral, and ventromedial PFC [40]. Although this was interpreted as a gender effect involving parts of the brain that may be the basis of inhibitory control functions, it is noteworthy that the ventromedial PFC (or OFC) is also a component of the brain’s reward circuit (suggesting a potential gender-based bias to high-caloric food cues). Furthermore, studies that have attempted to understand the neural basis of appetitive conditioning suggest that OFC function facilitates this particular form of learning, and that this probably plays a fundamental role in determining individual responses to rewarding stimuli such as high-caloric foods [41]. Although bilateral activations are typically observed in paradigms that use highly appetitive, high-caloric food cues, a primarily left lateralized network may play a key role in processing these images when participants are in a hunger state [42].

**Table 1:** fMRI studies of healthy subjects involving chocolate and high-caloric foods.

Authors	Subjects	Stimuli	Task	Results
Beaver *et al*., 2006 [26]	*n =* 12; 7 F/5 M	Vision: Appetizing, Disgusting, Bland, Non-Food	Passive Viewing	↑ L OFC, ventral striatum
Bohon *et al*., 2009 [43]	*n =* 20; 20 F 2 Groups: Emotional *vs*. NonEmotional Eaters	Taste and Vision: Chocolate milkshake, tasteless solution, or no solution visual shapes (cues)	Negative *vs*. Neutral mood induction	↑ L ventral ACC, thalamus across groups; ↑ L parahippocampal gyrus, ACC for emotional eaters in negative mood state during anticipation; ↑ L caudate, L pallidum, bilateral ACC during milkshake receipt in emotional eaters
Burger and Stice, 2012 [44]	*n =* 151; 74 M/77F Adolescents	Taste and Vision: Milkshake or tasteless solution Visual stimuli	Passive Viewing and Tasting	↓ bilateral putamen, R caudate, bilateral DLPFC, mid and anterior insula to milkshake receipt in frequent consumers
Burger and Stice, 2011 [39]	*n =* 39; 39 F Adolescents	Taste and Vision: Cues associated with milkshake reward, tasteless solution, or no solution	Viewing, consumption, and anticipation of food	Positive correlation between dietary restraint scores and R OFC, DLPFC milkshake > tasteless solution activations.
Coletta *et al*., 2009 [42]	*n =* 19; 19 F Restrained Eaters *n =* 9 Unrestrained Eaters *N =* 10	Vision: Highly palatable, moderately palatable, and non-food images	View images Before/after Satiety	↓ R STG, L parahippocampal gyrus, L putamen, L middle frontal gyrus (part of DLPFC) in unrestrained eaters; ↑ cerebellum, L MFG, L DLPFC, L cingulate gyrus, R IFG, R precuneus, L parahippocampal gyrus in unrestrained during fed state
Evero *et al*., 2012 [25]	*n =* 30; 17 M/13 F	Vision: High energy low energy Non-food items	Passive viewing after rest and exercise	↓ insula, ↑L precuneus activity to high-caloric foods after exercise
Frank *et al*., 2010 [27]	*n =* 12; 12 F	High-caloric, Low-caloric, Non-food pictures	Attend to pictures and imagine eating the food. 2 Sessions: Late follicular and Mid-late luteal phase	↑ R NAc, R amygdala, R hippocampus in follicular compared to luteal phase; ↑ R lateral OFC, L mid cingulum in luteal relative to follicular phase
Frank *et al*., 2010 [28]	*n =* 12; 12 F	High-caloric, Low-caloric Non-food stimuli	Food and non-food 1-back tasks; control task	↑ OFC, insula, occipital lobe, anterior and posterior cingulate cortex, thalamus, superior frontal lobe
Goldstone *et al*., 2009 [29]	*n =* 20; 10 M/10 F	Vision: High-caloric, low-caloric, non-food, and blurred images	Rate how appealing each image is during both fasted and fed states	↑ ventral striatum, amygdala, anterior insula, medial and lateral OFC when fasted
Killgore and Yurgelun-Todd, 2006 [45]	*n =* 13; 13 F	Vision: High calorie, low calorie, non-edible (utensils)	Attempt to remember images for later recognition test	↑ R lateral OFC with greater positive affect; ↑ medial OFC, subcallosal anterior cingulate gyrus, and posterior insula with greater negative affect
Killgore *et al*., 2003 [19]	*n =* 13; 13 F	Vision: High calorie, Low calorie, Non-edible (utensils)	Remember images for later recognition test	↑ bilateral mPFC, DLPFC, thalamus, R cerebellum, middle occipital gyrus, medulla
Kringelbach *et al*., 2003 [4]	*n =* 10; 10 M	Taste: Chocolate milk, tomato juice, tasteless solution	Passive tasting before and after satiety	↓ L OFC with satiety to tomato juice and chocolate milk, but no change with foods that were not consumed during the meal
Kroemer *et al*., 2013 [21]	*n =* 26; 13 M/13 F Fasting ghrelin levels measured	Vision: High palatable Low palatable food	Passive viewing	↑ bilateral middle and superior occipital/temporal gyrus, fusiform, caudate, pallidum, midbrain, rolandic operculum, amygdala, thalamus, anterior cingulate gyrus, hypothalamus
McCabe *et al*., 2010 [46]	*n =* 45; 21M/24F Citalopram *n =* 15 Reboxetine *n =* 15 Placebo *n =* 15	Taste and Vision: Liquid chocolate, liquid strawberry solution, tasteless solution; chocolate, gray control images	Rate stimulus pleasantness/unpleasantness after 7 days treatment with citalopram, reboxetine, or placebo	↑ ventral striatum, cingulate, mid OFC ↓ ventral striatum, ventral medial OFC to chocolate with citalopram ↑ activation to chocolate with reboxetine in medial OFC/frontal pole
Mehta *et al*., 2012 [20]	*n =* 23; 10 M/13 F	Vision: High-caloric, Low-caloric images	Attend to stimuli during deprived and various satiated sates	↑ bilateral amygdala positively associated with hunger scores and negatively associated with fullness in fasted state; ↑ R amygdala associated with greater hunger post-breakfast ↑ medial OFC positively associated with hunger scores in fasted state; ↑ medial OFC, L amygdala, L insula, bilateral NAc associated with food choice
Page *et al*., 2011 [30]	*n =* 21; 12 M/9 F	Vision: High-caloric, Low-caloric, Non-food images	Passive Viewing under euglycemic or hypoglycemic states	↑ striatum and insula during mild hypoglycemia; ↑ activity in ACC and ventromedial PFC correlated with higher blood glucose; ↑ insula and putamen correlates with high cortisol levels
Passamonti *et al*., 2009 [31]	*n =* 21; 11 M/10 F	Vision: Highly appetizing Bland food images No events	Indicate image position via button press	↑ ventral striatum, amygdala, ventral ACC
Piech *et al*., 2009 [36]	*n =* 8; 5 M/3 F	Vision: words Restaurant menu items High *vs*. Low attractiveness	Read menu item, Imagine dish in front of you, Rate how much you would like it during both hungry and satiated states	↑ amygdala, cerebellum to high attractive items; ↑ medial and lateral OFC to high attractiveness items when hungry
Rolls and McCabe, 2007 [2]	*n =* 16; 16 F Cravers *n =* 8 Non-cravers *n =* 8	Taste and Vision: Chocolate and condensed milk in mouth; dark and white chocolate images, grey visual cues	Rate pleasantness, intensity, and wanting for chocolate in each trial	↑ primary taste cortex, dorsal ACC, ↑ mid OFC, ventral striatum, DLPFC to chocolate in mouth; ↑ medial OFC in cravers *versus* non-cravers; ↑ ACC and pregenual cingulate cortex for cravers in sight and taste of chocolate condition; ↑ mid and medial OFC, ventral striatum for cravers to sight of chocolate
Schur *et al*., 2009 [32]	*n =* 10; 10 F	Vision: Fattening, non-fattening, and non-food object images	Remember what images were presented	↑ midbrain including ventral tegmental area, hypothalamus, L amygdala, L DLPFC, L OFC, R insula, striatum, thalamus, areas 17 and 18 of occipital lobe for fattening > non-food contrast; ↑ brainstem, R hypothalamus, L amygdala, R inferior frontal gyrus, insula, striatum, thalamus
Siep *et al*., 2009 [3]	*n =* 12; 12 F	Vision: High-caloric, Low-caloric, and Neutral images	Indicate palatability of foods and vividly imagine their tastes, color of neutral objects, or orientations of bars during food deprived and satiated states	Reduced inhibition of L medial PFC; ↑ fusiform, R medial OFC, R insula, L caudate putamen, PCC during hunger
Small *et al*., 2005 [5]	*n =* 11	Smell: Butanol, farnesol, lavender, and chocolate odors	Passive perception of odors Pleasantness/intensity ratings after each run	↑ medial OFC, perigenual cingulate to chocolate during retronasal administration; ↑ thalamus, R caudolateral OFC, R hippocampus, perisylvian and insular cortices for orthonasal administration
Smeets *et al*., 2006 [47]	*n =* 24; 12M/12 F	Taste: Chocolate milk, Eating solid chocolate	Taste chocolate milk during fasted and satiated states Indicate motivation to eat chocolate during scans	↑ L ventral striatum, L precentral gyrus, DLPFC, L dorsal striatum, anterior insula, OFC, medial OFC; ↓ inferior and superior parietal lobules, medial PFC for satiety in men; ↑ precentral gyrus, R superior temporal gyrus, ventral striatum; ↓ hypothalamus and amygdala for satiety in females

↑: Increased Activation; ↓: Decreased Activation.

Taken together, a great deal of research has identified brain structures that are activated by the sight of appetitive foods when healthy participants are satiated as well as when they are in fasted states, and these structures are believed to play a central role in reward processing. Many of these structures are also found to be active when patients suffering from substance abuse pathologies view drug stimuli (*i*.*e*., [48]), and further investigations may find ways to take knowledge obtained using fMRI and translate it into a clinically useful tool for treating various eating disorders.

### 3.2. Event-Related Potential Research (ERPs)

Although a great deal of research has been conducted using fMRI, there have been far fewer reports about the processing of high-caloric food cues by healthy participants using EEG/ERP technology (see Table 2). Although there appears to be a trend in this direction, there is ample room for growth in this area and findings obtained with this technique can provide insight into the real-time processing of these salient environmental cues.

Studies using the ERP technique have attempted to understand how the brain differentially processes high-caloric foods, low-energy foods, and non-food stimuli using male, female, and mixed gender samples. A recent study reported modulations of an early component occurring between 170 and 213 ms after stimulus presentation in response to high-caloric relative to low-caloric foods in an all-female sample, although no significant differences were observed within the male group. Source localization procedures indicated possible brain generators for this scalp-recorded brain electrical activity in posterior visual association areas (typically involved in object recognition and attention to salient stimuli), and in bilateral inferior frontal cortex [49]. Importantly, within the female group, modulation of this component was found to co-vary with body mass index values (BMI), despite the fact that their values were within the normal range. A later occurring positive component (309–371 ms) was also found to be greater in response to high-fat *versus* low-fat pictures, and its estimated source generator was found to localize to the ventromedial PFC in addition to dorsal and lateral prefrontal areas in the right hemisphere (an effect that resembles other findings obtained using similar techniques; [50]). These effects are significant, as they suggest that females are more likely to be influenced by the sight of high-caloric food cues early on in stimulus processing, which seems to corroborate gender effects found using fMRI [40]. Research has also found that perception of taste can be amplified based on whether a person is currently viewing high-caloric or low-caloric food stimuli. Using a novel design, a second recent study administered “electric taste” via delivery of an electrical current to different regions of the tongue while neural responses to presentations of food cue images were recorded with EEG equipment [51]. Several effects were identified in the early and later stages of stimulus processing. An early anterior effect was observed within 176–236 ms after high-caloric food stimulus presentation, which was source localized to the OFC (indicating that this stimulus was coded as having greater reward value). A later latency posterior effect was found between 357 and 500 ms from stimulus onset, which was source localized in the right insular cortex (which suggested that the stimulus had greater hedonic value). When the electrophysiological responses to taste stimuli administered shortly after high or low-caloric stimulus presentations were source localized, the timeline of early and late occurring events almost perfectly represented the anatomically defined gustatory processing stream. At around 100 ms after taste stimulus presentation, increased activation was observed in the insula and frontal operculum (primary taste cortex). At around 180 ms, increased activation was then observed in the OFC (which is part of the secondary taste cortex). Finally, activation appeared to return to the insula/frontal operculum at 360 ms (perhaps reflecting an attempt at multimodal integration within the central nervous system). Importantly, this degree of fine temporal resolution cannot be achieved with fMRI (although it could be reached by magnetoencephaloghy (or MEG) as this technique records changes in magnetic field strength that are produced when an electrical dipole is active). Despite the fact that MEG could also be used to acquire results similar to these, EEG equipment and its corresponding maintenance cost exponentially less than that of MEG, which could potentially bolster the initiation of future studies and subsequent replication of empirical findings obtained by them. Researchers who were interested in assessing the temporal dynamics of high-caloric stimulus processing have also found that electrophysiological components elicited after presentations of low-energy foods and non-food items showed amplitude modulations over the course of repeated testing, yet activations seen in response to high-energy foods remained the same and did not habituate [52]. Finally, one study has also found differences in ERP responses to appetitive food cues based on whether participants were hungry or satiated, with exaggerated responses being observed for 2 positive amplitude components occurring between 170–210 ms and 270–310 ms during hunger states (perhaps indicating that an early attentional bias to such cues was present) [53]. Importantly, these responses were found to be unique to states of hunger, as there were no condition-based differences in response to other affective stimulus presentations.

Other research that looked at late-occurring ERP components found that a potential called the Late Positive Potential (LPP) increased in amplitude after presentations of highly appetitive pictures relative to neutral control stimuli [54]. Additionally, greater attentional biases towards local *versus* global features as measured by reaction times (RT) were also successfully predicted by LPP voltages, suggesting that the stimulus was highly salient and that it received special processing. This component has also been elicited to a greater degree when appetitive foods were presented to participants in a deprived state [55] and when high-caloric (dessert) pictures were presented to restrained *versus* unrestrained eaters who were told that certain foods would be available or unavailable to them. In the latter case, restrained eaters showed less of an LPP response to available food cues, which may have reflected a mobilization of cognitive control strategies by members of this group [56].

Taken together, ERP research has consistently identified both early and late-occurring effects in response to high-caloric food cues, and the amplitude and polarity of some of these components are modulated by within-subjects factors such as hunger state or restrained *versus* unrestrained eating style. In addition to using the classic approach of reporting the topographic location of the ERP components at the level of the scalp, recent research has also implemented source localization procedures in an effort to estimate the neural generators of these electrical potentials, with their solutions appearing similar to those results obtained using fMRI tasks that assessed neural responses to high-caloric foods. The utility of using ERPs to study normal and abnormal processing of highly appetitive food cues is an area of research that is still developing, but it has great potential for informing scientists about how human beings process these biologically relevant cues in real time, as well as how this processing maps on to classic descriptions of sensory processing streams identified by neuroanatomy.

**Table 2:** EEG/ERP studies using chocolate and high-caloric food stimuli with healthy participants.

Authors	Subjects	Stimuli	Task	Results
Asmaro *et al*., 2012 [1]	*n =* 26; 26 F Chocolate Cravers (*n =* 14) NonCravers (*n =* 12)	Vision: Chocolate and bland food images	Passive viewing with satiety between sessions	EAP (250–350 ms); LPP (360–560 ms); AN (100–250 ms)
Blechert *et al*., 2010 [56]	*n =* 40; 40 F Restrained Eaters (*n =* 19) *vs*. Non-restrained Eaters (*n =* 21)	Vision: High-caloric, pleasant, neutral, unpleasant pictures	Passive viewing of available and unavailable foods	↓ LPP (300–700 ms) to available food cues in restrained eaters
Gable and Harmon-Jones, 2010 [54]	*n =* 30; 19 M/11 F	Vision: Appetitive (desserts) neutral (rocks), Navon stimuli	View appetitive and neutral pictures; decide whether Navon stimuli contained either the letter *T* or letter *H*	↑ LPP (500–1000 ms) to appetitive cues
Kemmotsu and Murphy, 2006 [57]	17 female restrained eaters 18 female unrestrained eaters	Smell: Chocolate and non-food odors	Ignore or attend to chocolate and non-food odors	↑ N1P2 in unrestrained to chocolate odor in ignore condition; ↑ N1P3 in unrestrained to chocolate odor for attend relative to ignore condition
Lietti *et al*., 2012 [52]	*n =* 21; 10 M/11 F	Vision: High energy, low energy, and non-food stimuli	Decide whether stimulus is a food or non-food item	No difference is VEP topographies when high energy foods repeatedly presented; Source localization indicates generators in prefrontal and middle temporal cortices
Ohla *et al*., 2012 [51]	*n =* 14; 9 M/5 F	Vision, Taste: High-caloric, low-caloric, and non-food images; taste stimuli delivered by electrical stimulation of tongue	Categorize food and non-food images; evaluate taste pleasantness and intensity	↑ source strength in R insular gyrus, FOP transition, L FOP, L middle frontal gyrus, R parahippocampal gyrus (92–174 ms) when high-caloric images preceded taste; ↑ medial orbitofrontal gyrus, ACC, left superior and middle frontal gyrus, parahippocampal, fusiform (176–236 ms) when high-caloric images preceded taste; ↑ R middle frontal gyrus, R insula, parietal operculum, postcentral gyrus, R occipital gyrus (357–500 ms) when high-caloric viewed before taste stimulation
Stockburger *et al*., 2009 [55]	*n =* 32; 16 M/16 F	Vision: Appetizing food, pleasant, unpleasant, neutral, and flower images	View images in food deprived and satiated states	↑ positivity to food images over posterior electrode sites (60–300 ms); ↑ negative potential to food at occipito-temporal sensors (more left-sided) and a centro-frontal positivity during deprived state (300–350 ms); ↑ positive potential at parietal sensors (polarity reversed at frontal channels) to food during deprived state (450–600 ms)
Stockburger *et al*., 2008 [53]	*n =* 16; 16 M	Vision: Appetizing food, Pleasant, Unpleasant, Neutral images	View images during food deprived and satiated states	↑ posterior positivity to food (170–210 ms and 270–310 ms) during hungry state
Toepel *et al*., 2012 [49]	*n =* 24; 12 M/12 F	Vision: High fat, Low fat Non-food images	Decide whether stimulus was a food or non-food item	↑ VEP for women, which interacted with BMI values (170–213 ms); Source localization found potential generators in ventromedial PFC, posterior middle temporal cortex, superior parietal lobe of left hemisphere; ventromedial PFC, anterior temporal cortex, and inferior parietal lobe
Toepel, *et al*., 2009 [50]	*n =* 24; 12 M/12 F	Vision: High fatLow fat Non-food images.	Decide whether stimulus was a food or non-food item	↑ VEP to high fat food images between 166–230 and 309–371 ms; Source localization found lateral occipital, superior temporal, left postcentral gyrus (166–230 ms); Source localization found greater L occipito-temporal cortex, L inferior parietal lobule, L dorsal frontal, R ventromedial PFC, R dorsal and lateral PFC (309–371 ms)

↑: Increased Activation, ↓: Decreased Activation.

## 4. Research with Chocolate as a Target Stimulus

Although this discussion has focused on high-caloric food cues up until the current point, special mention should be made about the unique effects reported by researchers who have used chocolate stimuli as a target of interest. Chocolate is unique in the sense that it is commonly used as a positive reinforcer for desirable behaviour by parents during their offspring’s early life and adolescent years, and this is especially true in Westernized societies. Some earlier scientific work that utilized the positron emission tomography (PET) technique focused on changes in regional cerebral blood flow within the brain as the hedonic value of chocolate decreased as a function of increased consumption, where reductions in bilateral insula activation were observed when chocolate became less rewarding [58]. By looking at neural responses to chocolate, researchers can assess how the brain’s reward system is activated by its sight as well as how activation in this circuit changes as a function of satiety. Results from studies such as these can help scientists understand differences between individuals who are cravers or non-cravers, and the neural correlates of craving can also be determined. Furthermore, by using techniques such as fMRI and ERPs, hemodynamic and electrophysiological responses to chocolate cue reactivity and consumption can also be delineated.

### 4.1. Chocolate Cue Reactivity and fMRI

Research on chocolate stimulus processing that used fMRI has found a number of neural effects that appear to be modulated by the sight, smell, taste, anticipation of receiving, and consumption of the substance (*i*.*e*., [2,5,13,39,43,44,46,47,59,60]). Regarding neural activations to the cueing and consumption of chocolate, the right OFC and bilateral DLPFC were found to respond when participants anticipated and received a chocolate milkshake *versus* a tasteless solution [39]. These BOLD responses were found to positively correlate with self-reported eating restraint scores, which suggest that greater activation of these structures during anticipation for and receipt of chocolate-based food rewards may be indicative of problematic food consumption habits. In another study that used a chocolate milkshake paradigm, female emotional eaters were found to have increased left ventral anterior cingulate cortex activity when they anticipated the receipt of chocolate milkshake during a negative mood state. However, increased left caudate and pallidum responses were subsequently observed after the milkshake was actually administered [43]. This was thought to suggest that female emotional eaters may be especially responsive to the rewarding properties of food (in this case, the taste of chocolate milkshake), therefore making them vulnerable to overeating. Another study also found activations in the right lateral OFC, dopaminergic midbrain, and right posterior superior temporal sulcus when participants anticipated the acquisition of a chocolate prize after winning a modified “wheel-of-fortune” game [60], once again confirming that chocolate can activate structures within the brain’s reward system. Furthermore, studies looking at the effects of tasting chocolate have found activations of the medial OFC and ventral striatum [2,47], thus providing further evidence that the OFC plays a critical role in signaling hedonic responses and in acting as a multimodal integration system for the processing of appetitive stimuli [2,61]. Interestingly, activation of the left OFC to the taste of chocolate milk has also been found to decrease with levels of satiety, with a significant negative correlation being found between these two variables [4]. Related to the finding that the OFC may play a major role in signaling incentive value for rewarding environmental stimuli, pharmacological research has found that the selective serotonin reuptake inhibitor citalopram caused a decrease in medial OFC and ventral striatum reactivity to the sight of chocolate while reboxetine (a norepinephrine reuptake inhibitor) increased cue-reactivity in healthy subjects [46], thus demonstrating a novel use for chocolate stimuli in studies that attempt to understand the neural basis of depressive symptoms (*i*.*e*., anhedonia) which also involve abnormalities in patients responding to appetitive stimuli (see [41] for a review). One study has also reported that participants were alleviated from an experimentally induced negative mood state after eating chocolate, and that chocolate craving increased as negative mood worsened [62].

In summary, a diversity of fMRI research has been conducted using chocolate stimuli and healthy participants, with studies ranging from investigations of its effects on mood states to its ability to engage neural regions implicated in reward processing and conditioning. This research has facilitated insights into the neural basis of learning, satiety, mood regulation, reward, and also allows us to make inferences about the neural correlates of pathological conditions (*i*.*e*., overeating, anhedonia, and addiction).

### 4.2. Chocolate Cue Reactivity and ERPs

In relation to work that has been done using chocolate and fMRI with healthy participants, there have been almost no ERP studies done with similar paradigms. In this section, we will present findings from three studies (one of which we published ourselves), in an effort to show the utility of using chocolate stimuli and the ERP technique in tandem, as well as how ERP findings can be interpreted in light of fMRI findings regarding chocolate and the brain.

One study attempted to describe the differential processing of chocolate odors in a sample of restrained eaters relative to a control group [57]. While presenting these participants with chocolate and non-food odors and giving them instructions to attend to or ignore the smell, it was found that the restrained eaters showed an attenuated evoked-potential response to the chocolate odor only within the “attend” condition. The authors suggest that restrained eaters may have been attending to the chocolate odor when they were supposed to be ignoring it, and that they could also have been employing a cognitive control strategy to reduce their responsivity. Another study found that chronic use of cocoa-flavored flavanols altered the amplitude of the steady state visually evoked potential (SSVEP) when participants were tested before and after a 30-day cocoa flavanol consumption period. This was thought to reflect an improvement in working memory efficiency [63]. Here, the amplitude of the SSVEP was found to decrease over posterior parietal electrode sites during the encoding, retention, and retrieval phases of memory when medium and high doses of the flavanols were administered. Based on results obtained from these two studies, chocolate stimulus processing may indeed be a useful topic of investigation for studies aimed at understanding multimodal stimulus processing, as well as the effects of chocolate on human cognition. The exquisite temporal resolution of the ERP technique makes it ideal for studying the effects of chocolate on cognition, and future research may wish to assess different cognitive effects before and after chocolate consumption across gender and within samples of patients suffering from various pathological conditions.

**Figure 1:**
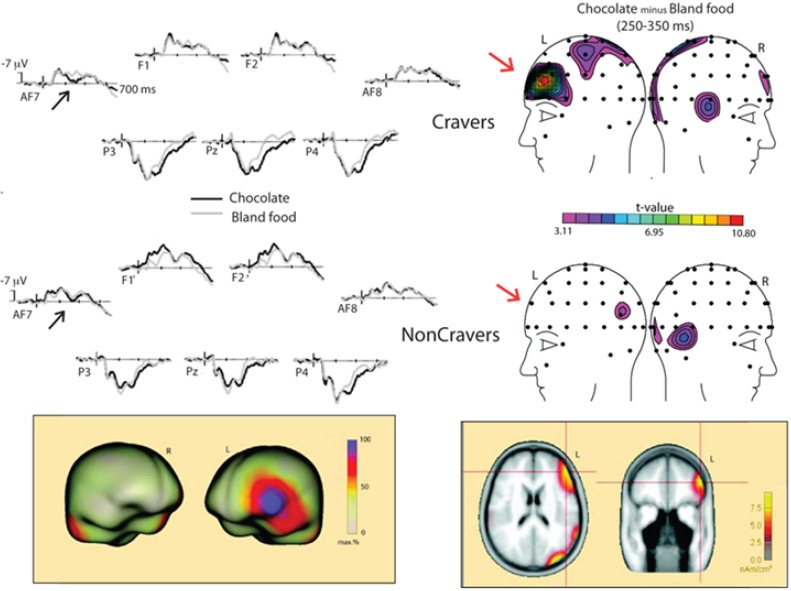
ERP waveforms and scalp topography associated with chocolate cravers viewing chocolate and bland food stimuli (Asmaro *et al*., 2012). The bottom part of the figure illustrates the brain surface and source localization results done with the AP (250–350 ms) effect that differentiated chocolate cravers from non-cravers [1].

Before concluding this section, we would like to summarize a recent ERP study we conducted that explored chocolate stimulus processing among samples of participants who were either high or low in trait chocolate craving [1]. In this study, we had participants view short sequences of chocolate versus bland food pictures. After they viewed 100 stimulus presentations of each stimulus category, they took a break and consumed chocolate until satiated. The same number of stimuli were presented in a second session, and ERP responses to both categories of stimuli across both sessions were recorded. We found a significantly greater modulation for an early positive potential in the craver group relative to the non-cravers when they viewed chocolate stimuli (250–350 ms time window, an effect called anterior positivity or AP (see Figure 1). Importantly, this effect did not disappear following the satiety manipulation (which may constitute a biomarker for trait chocolate craving). Please note that the AP effect was not isolated by between-group comparisons. Rather, the effect was found when comparing the chocolate cue and neutral cue responses within the high-craver and low-craver groups (which helps control for inequalities in baseline neutral cue responses across groups that could ultimately confound a between-groups analysis). An additional finding in the study was that the low-craver group responded differently to the chocolate stimuli in the first session, with an earlier, negative polarity difference, evident between 100 and 250 ms following stimulus onset. The effect had a different scalp topography over the middle frontal region (called anterior negativity or AN; see Figure 1). Given that chocolate is frequently used as a positive reinforcer in Western societies, we believe that our low-craver group may have automatically regulated conditioned hedonic responses to the sight of chocolate cues in the first session using top–down inhibitory control, while this process was not elicited in the second session (perhaps due to recent reward receipt). Importantly, this effect appeared to replace the AP to chocolate stimuli seen in the craver group during the first session, and the fact that it occurred earlier in time was thought to indicate that the earlier occurring AN may have caused modulation of AP amplitude for non-cravers (which may have ultimately influenced subjective craving). Across both groups for both sessions, a late positive potential (LPP) effect in the 360–560 ms window was also observed, indicating that both groups deployed attentional resources towards this affective stimulus and that this did not diminish with satiety. In addition to looking at differences in scalp-recorded electrical activity, we also used source localization techniques, with the hope that the frontal response seen in the craver group to chocolate stimuli would source localize to the OFC. Using the SSLOFO (Standardized Shrinking LORETA-FOCUSS) technique on the chocolate minus neutral difference wave, a left lateral OFC source was found to be a possible generator of this positive going AP effect, similar to previously cited fMRI reports (therefore supporting the idea that the OFC is a structure that processes appetitive stimuli and facilitates decision making that guides observable behavior; [13,64]). These results, in addition to the results reported in three recent studies that assessed the impact high-caloric stimuli have on human electrophysiology [49,50,51] show that the ERP technique can provide insightful information regarding the processing of chocolate and other high-caloric cues. Also, the technique may help researchers better understand how chocolate and other food stimuli are processed within the gustatory and olfactory systems (including how flavor intensity is coded by their association cortices). Because of the limitations of the ERP technique’s spatial resolution and because of the limited number of studies that employed source localization to plot scalp-recorded components in brain space, more work will need to be done in order to verify and replicate the source localization results as they only provide estimates regarding neural regions that potentially become active during cue presentation. Still, we believe that these early ERP studies of chocolate processing within the human brain may provide a much needed foundation for future research that attempts to better consolidate fMRI findings with those found using other techniques.

## 5. Factors Involved in the Modulation of Food-Elicited Neural Reponses

Although this review has focused on research that attempts to highlight neural responses to high-caloric food and chocolate stimuli, it is important to note that many factors may influence observations seen using either fMRI or ERP food-cue paradigms. As can be seen in the preceding text, many factors including (but not limited to) affective state [45], discrete personality traits such as self-directedness [65], exercise prior to viewing experimental stimuli [25], level of hunger/satiety [1,30], and reward sensitivity [26] have been shown to influence how various samples of participants respond to these cues during experimental tasks. In order to fully appreciate how the brain responds to foods with high-caloric content, the potential moderating and mediating effects of these factors need to be strictly accounted for when paradigms used for such investigations are employed. Although research has examined the effects of some of these sources of influence, replication of the effects cited in these reports would be especially beneficial. In addition, further exploration of personality, environmental, affective, and clinical variables that could modulate the neural processing of food cues would help to better align research findings with the diverse variety of responses typically observed in human beings. By properly accounting for the breadth of factors that may modulate brain responses to highly appealing food cues, a more accurate picture of how the human brain uniquely processes essential nutritional information in our environments can be cataloged. Undertaking this line of investigation could lead to the development of a regression-based model that allows for the accurate prediction of high-caloric food cue responses in healthy and pathological human subjects by taking into account the wide array of circumstances that could act on an individual at any given point in time.

## 6. Conclusions

How the brain responds to highly appetitive foods has been a fascinating research area for many scientists, and, over the course of the last decade, there has been tremendous advances in understanding the neural correlates of appetitive food cue processing. Given the special status of chocolate in Western culture, studies have also been conducted using this stimulus as a target, and its presentation has been found to activate many of the same brain regions that are activated by high-caloric foods and other reward stimuli. Electrophysiology research has also found a number of ERP components that seem to be elicited specifically by high-caloric food pictures and chocolate stimuli, although more research will need to be done in order to fully understand the processing of these stimuli in the temporal domain. As a result of advances in computer technology, sophisticated source localization procedures can now be used to localize electrical activity recorded at the scalp, therefore facilitating comparisons with fMRI results. Using chocolate and high-caloric stimuli may provide useful information about pathological and normal brain processes, and understanding how chocolate consumption affects cognition is still in its infancy. Although this list of potential research directions is not exhaustive, it nonetheless conveys the notion that there is still much to be learned regarding the human processing of appetitive stimuli while highlighting the converging findings that have been obtained through multiple methodologies that will enrich our knowledge regarding this universal human neurocognitive response.
